# Role of Adaptive Immunity in Alcoholic Liver Disease

**DOI:** 10.1155/2012/893026

**Published:** 2011-09-07

**Authors:** Emanuele Albano

**Affiliations:** Department of Medical Sciences and Interdisciplinary Research Centre for Autoimmune Diseases (IRCAD), University “Amedeo Avogadro” of East Piedmont, Via Solaroli 17, 28100 Novara, Italy

## Abstract

Stimulation of innate immunity is increasingly recognized to play an important role in the pathogenesis of alcoholic liver disease (ALD), while the contribution of adaptive immunity has received less attention. Clinical and experimental data show the involvement of Th-1 and Th-17 T-lymphocytes in alcoholic hepatitis. Nonetheless, the mechanisms by which alcohol triggers adaptive immunity are still incompletely characterized. Patients with advanced ALD have circulating IgG and T-lymphocytes recognizing epitopes derived from protein modification by hydroxyethyl free radicals and end products of lipid-peroxidation. High titers of IgG against lipid peroxidation-derived antigens are associated with an increased hepatic production of proinflammatory cytokines/chemokines. Moreover, the same antigens favor the breaking of self-tolerance towards liver constituents. In particular, autoantibodies against cytochrome P4502E1 (CYP2E1) are evident in a subset of ALD patients. Altogether these results suggest that allo- and autoimmune reactions triggered by oxidative stress might contribute to hepatic inflammation during the progression of ALD.

## 1. Introduction

According to the World Health Organization, alcohol-related diseases are the third cause of death and disability in most well-developed countries and a leading cause of disease in the developing countries in Eastern Europe, Central and South America, and East Asia [1]. Although several organs are injured by ethanol, alcoholic liver disease (ALD) is the most common medical consequence of excessive alcohol intake accounting for about 70% of the recorded mortality [2]. Thus, understanding the mechanisms responsible for alcohol liver injury has a relevant clinical and social impact. Current view indicates that multiple factors including oxidative stress, endoplasmic reticulum stress, metabolic alterations, and interferences with the transduction of intracellular signals are involved in ALD pathogenesis [3]. Furthermore, emerging evidence suggests that chronic inflammation represents the driving force in the evolution of alcohol liver injury. In this context, a large number of studies have investigate the role of innate immunity in ALD ([4–8] for recent review), while the contribution of adaptive immunity to alcohol-induced hepatic inflammation has received much less attention. This paper will give an overview of the possible implications of adaptive immunity in the inflammatory processes associated with ALD. 

Early studies have shown that liver inflammatory infiltrates in alcoholic hepatitis and active alcoholic cirrhosis contain both CD8^+^ and CD4^+^ T-lymphocytes [9]. In either chronic alcohol-treated mice or alcohol abusers liver-infiltrating T cells express an activation/memory phenotype and respond to T-cell receptor stimulation by producing Th-1 cytokines such as interferon-*γ* (IFN-*γ*) and TNF-*α* [10, 11]. A Th-1 cytokine pattern is also evident in peripheral blood T cells from active drinkers with or without ALD [12]. In agreement with these observations, Paronetto [13] reported the presence in ALD patients of circulating antibodies targeting alcohol-altered autologous hepatocytes. Hyperproduction of polyclonal gamma globulins is also frequent in alcohol abusers in association with tissues deposition of IgA [14]. Moreover, ALD patients do not rarely have signs of auto-immunity consisting in increased titers of circulating antibodies directed against non-organ-specific and liver-specific autoantigens [15]. In particular, antiphospholipid antibodies can be observed in up to 80% of patients with alcoholic hepatitis or cirrhosis, but are not infrequent in heavy drinkers with milder liver damage [16, 17]. A further evidence supporting the implication of adaptive immunity in ALD comes from the recent demonstration that IL-17-producing T helper (Th-17) lymphocytes are evident in hepatic inflammatory infiltrates of patients with alcoholic hepatitis/cirrhosis in concomitance with an increase in IL-17 plasma levels [18]. The implication of Th-17 T-cells in ALD is particularly important considering the increasing importance ascribed to these cells in the pathogenesis of several chronic inflammatory diseases including viral hepatitis B and C and primary biliary cirrhosis [19].

## 2. Role of Oxidative Stress in Alcohol-Induced Immune Reactions

The mechanisms by which alcohol triggers adaptive immunity are still incompletely characterized. Pioneering study by Israel and colleagues [20] has shown that the adducts originating from acetaldehyde binding to hepatic proteins cause the production of specific antibodies when injected into experimental animals. The presence of antiacetaldehyde antibodies has been subsequently confirmed in rats chronically exposed to alcohol as well as in alcoholic patients [21, 22]. Guinea pig immunization with acetaldehyde-modified hemoglobin followed by alcohol feeding reproduces several features of alcoholic hepatitis [23]. However, the interest for acetaldehyde-induced immune responses is hampered by the uncertainty regarding identity of the antigens involved and by the low specificity for ALD of anti-acetaldehyde antibodies [24]. Subsequent studies demonstrate that another metabolite of ethanol, that is, hydroxyethyl free radical (HER), produced during cytochrome P4502E1- (CYP2E1-) dependent ethanol oxidation, can interact with proteins generating antigens distinct from those derived from acetaldehyde [25]. Anti-HER IgG are detectable in chronically ethanol-fed rats as well as in alcohol abusers [26, 27]. Human anti-HER IgG are recognized as main antigen HER-modified CYP2E1 [28], and their presence strictly correlates with CYP2E1 activity [29]. 

Oxidative stress is one of the feature of alcohol hepatotoxicity and significantly contributes to liver injury [30]. One of the consequence of oxidative stress is the stimulation of lipid peroxidation with the generation of wide array of reactive lipid breakdown products including aldehydes such as malondialdehyde (MDA), 4-hydroxynonenal (4-HNE), and lipid hydroperoxides that are readily detectable in the serum and the liver of ALD patients and alcohol-fed rodents [30]. Many lipid peroxidation products are highly reactive and by interacting with cellular constituents generate antigenic products that have been implicated in the stimulation of immune responses associated with atherosclerosis and several autoimmune diseases [31, 32]. We have observed that a large proportion (55–70%) of the patients with advanced ALD (alcoholic hepatitis and/or cirrhosis), but not heavy drinkers with fatty liver only, have elevated titers of circulating IgG against proteins adducted by MDA, 4-HNE, and oxidized arachidonic acid [33]. In about 35% of these patients, the presence of anti-MDA antibodies is associated with the detection of peripheral blood CD4^+^ T cells responsive to MDA adducts, indicating the capability of lipid oxidation antigens to trigger both the humoral and cellular branches of adaptive immunity [34]. At present, the chemical identity of the different antigens involved is still incompletely characterized. Studies by Tuma and Thiele have shown that the reaction between MDA, acetaldehyde, and lysine *ε*-amino groups generates condensation products named malondialdehyde-acetaldehyde adducts (MAA) that not only are highly immunogenic, but also can stimulate inflammation [35]. MAA adducts have been detected in the liver of ethanol-fed rats, and their formation is responsible for the increased titers of IgG recognizing MAA-modified proteins in patients with advanced ALD [36]. Considering that appreciable amounts of acetaldehyde and MDA are generated in the liver during alcohol intoxication, it is well possible that MAA adducts might significantly contribute to immune response in ALD. It is noteworthy that rodents and humans have circulating natural antibodies, mainly of the IgM class, targeting oxidation-derived epitopes including MDA and MAA adducts [37, 38]. These natural antibodies display protective action against atherosclerosis by scavenging oxidized LDL and preventing inflammation [37]. In our hands, the antibody responses observed in ALD patients specifically involve only IgG, while IgM against MDA and MAA adducts do not differ from those in healthy controls [33, 36]. This suggests that the extensive lipid peroxidation caused by alcohol abuse might overcome the scavenging capacity of natural IgM antibodies and, in combination with inflammatory stimuli, favors the activation of B- and T-cell clones recognizing a variety of oxidation-derived antigens. Oxidative stress also likely accounts for the development of ALD-associated antiphospholipid antibodies, since they recognize as antigens oxidized phospholipids, namely, oxidized cardiolipin and phosphatidylserine [39, 40]. Interestingly, the presence of IgG targeting lipid-peroxidation-derived antigens is evident in patients with chronic hepatitis C (CHC) consuming moderate amounts of alcohol and increases in a dose-dependent manner with alcohol intake [41]. This is consistent with recent observations about the synergic action of ethanol and hepatitis C virus in promoting oxidative stress within the hepatocytes [42].

## 3. Mechanisms Possibly Involved in the Recruitment of Adaptive Immunity in Alcoholic Liver Disease

It is known since long time that excessive alcohol consumption affects the innate and adaptive immunity increasing the susceptibility to infections and compromising tissue response to injury [5]. In particular, both acute and chronic alcohol intakes depress antigen presentation by monocytes and dendritic cells, affect the expression costimulatory molecules, and reduce T-cell proliferation [5]. It is possible that, during the evolution of ALD, the production of proinflammatory cytokines/chemokines by Kupffer cells and NKT lymphocytes might overcome alcohol-dependent immune depression promoting the response of intraportal lymphoid follicles to antigens derived from oxidatively damaged hepatocytes. Alcohol-induced oxidative stress can specifically facilitate this process through several mechanism. In either rodents and humans, chronic alcohol exposure increases the levels of circulating bioactive oxidized phospholipids able to interact with plated activating factor (PAF) receptors and to stimulate inflammation [43]. Furthermore, oxidized lipid and protein adducted by lipid peroxidation end products act as danger-associated molecular patterns (DAMPs) and are capable of activating inflammatory and immune cells through the interaction with pattern recognition receptors (PRRs) such as scavenger receptors (SRA-1,2, CD36, SR-B1, and LOX-1) and toll-like receptors (TLR-4) [44]. In particular, CD36 efficiently recognizes free and protein-bound oxidized lipids favoring their internalization by macrophages and antigen-presenting cells with their subsequent presentation to immune cells [44]. The interaction between CD36 and TLR-4 also promotes proinflammatory activation of macrophages and vascular endothelial cells in response to oxidation-specific epitopes [44]. In the liver, the presentation of oxidative-stress-derived antigens by hepatic stellate cells (HSCs) might represent an additional pathway for CD4^+^ and CD8^+^ T-cell activation, as human HSCs are efficient professional antigen presenting cells [45] and have the capacity to internalize oxidatively modified proteins through CD36 [46]. According to this scenario, time course experiments performed in enteral alcohol-fed rats show that the hepatic mRNAs expression of Th-1 cytokines (TNF-*α*, IL-12) has a biphasic pattern peaking after 14 days of alcohol feeding and then rising again after 35 days [47]. Oxidative stress is evident already after few days of alcohol exposure, but lipid-peroxidation-derived antibodies are evident in concomitance with the late increase cytokine production [47] and their formation is prevented by the antioxidant N-acetylcysteine [48]. Moreover, stimulation of B cells through Toll-like receptor 9 (TLR-9) has been implicated in causing hyperimmunoglobulinemia in patients with alcoholic cirrhosis [49]. The immune responses in ALD might be influenced by alterations of the immunoregulatory mechanisms. Hepatic steatosis and oxidative stress lower regulatory T-cells (Tregs) population in the liver [50]. This might impact on B- and T-cell activation by oxidation-specific epitopes, as, during the evolution of atherosclerosis, Tregs impairment favors lymphocyte responses against oxidized LDL [51]. Finally, further stimuli to adaptive immunity in ALD might involve osteopontin and the adipokines leptin and adiponectin. Osteopontin is a cytokine produced by many cell types, including macrophages and T-lymphocytes, that promotes macrophage and T-cell activation, stimulates lymphocyte Th-1 and Th-17 differentiation, and induces B-cell proliferation and antibody production [52, 53]. Increased osteopontin is a feature of several inflammatory and autoimmune diseases, where it directs the recruitment of autoreactive T cells and lymphocyte Th-1 and Th-17 differentiation [53]. An increased liver production of osteopontin correlates with the extent of inflammation in alcohol-fed rodents [54], while hepatic osteopontin mRNA expression is higher in patients with alcoholic hepatitis than in heavy drinkers with fatty liver only [55]. Leptin and adiponectin originating from the adipose tissue have immunoregulatory functions: leptin stimulates lymphocyte survival and proliferation favoring Th-1 reactions, while adiponectin downregulates macrophage activity and B- and T-cells proliferation [56]. Alcohol influences adipocyte production of adipokines increasing serum leptin in patients with alcoholic liver disease (ALD) [57] and lowering adiponectin secretion [58]. 

Little is known about the origin of antiphospholipid antibodies often associated with ALD. In the recent years, increasing evidence has linked defects in the disposal of apoptotic cells with the development of antiphospholipid antibodies [59, 60]. In particular, phagocytosis of apoptotic bodies by immature dendritic cells in an inflammatory context can lead to the presentation of apoptotic cell-derived self-antigens to T-lymphocytes [59, 60]. We have observed that antiphospholipid antibodies from the sera of ALD patients bind to apoptotic, but not to living cells, by specifically targeting oxidized phosphatidylserine expressed on the cell surface [40]. This is consistent with the detection of oxidized phosphatidylserine on surface of apoptotic bodies [61] and suggests that ALD-associated antiphospholipid antibodies may originate from alterations in the disposal of apoptotic hepatocytes. Indeed, growing evidence points to the importance of apoptosis-derived antigens as targets of antiphospholipid antibodies [62]. Hepatocyte apoptosis is one of the consequence of chronic alcohol intake [30], and an increase in apoptotic bodies is well evident in liver biopsies of ALD patients [63]. At the same time, alcohol impairs the capacity of neighboring hepatocytes to dispose apoptotic cells through asialoglycoprotein receptor-mediated phagocytosis [64] and primes Kupffer cells to produce TNF-*α* and IL-6 when exposed to apoptotic bodies [65]. 

The presence of both non-organ-specific and liver-specific autoantibodies is a common feature in ALD. Among these latter, we have reported that alcohol-fed rats as well as about 40% of the patients with advanced ALD develop circulating IgG directed against CYP2E1 [66, 67]. Anti-CYP2E1 autoantibodies from ALD patients are similar to those associated with halothane hepatitis and recognize at least two distinct conformational epitopes on the molecule surface, in a position compatible with the targeting of CYP2E1 present on the outer layer of the hepatocyte plasma membranes [68]. The development of anti-cytochrome P450 (CYP) isoenzymes is rather common in liver diseases such as type-2 autoimmune hepatitis, drug-induced hepatitis, and hepatitis C [69]. In drug-induced hepatitis, it has been postulated that the binding of reactive metabolites to CYPs promotes both humoral immune responses against the drug-derived epitope(s) and, at the same time, favors the activation of normally quiescent autoreactive lymphocytes recognizing the native CYP molecules [69]. ALD patients with anti-HER antibodies have a 4-fold increased risk of developing anti-CYP2E1 autoreactivity as compared to patients without anti-HER IgG [67]. This indicates that CYP2E1 alkylation by HER is involved in the development of ALD anti-CYP2E1 autoantibodies. Nonetheless, additional factors might favor the breaking of self-tolerance in ALD. We have observed that a polymorphism (Thr^17^ → Ala substitution) in the cytotoxic-T-lymphocyte-associated antigen-4 (CTLA-4) gene increases by 3.8-fold the risk of developing anti-CYP2E1 IgG without influencing the formation of anti-HER antibodies [67]. ALD patients having both anti-HER IgG and mutated CTLA-4 show a prevalence of anti-CYP2E1 autoreactivity 23-fold higher than those negative for both these factors [67]. CTLA-4 is a membrane receptor expressed by activated T-lymphocytes and by CD25^+^ CD4^+^ Tregs that downmodulates T-cell-mediated responses to antigens [70]. Accordingly, in humans, CTLA-4 genetic polymorphisms are risk factors for several autoimmune diseases, including primary biliary cirrhosis and type-1 autoimmune hepatitis [71]. More recently, Thiele and coworkers have shown that mice immunization with MAA-modified liver cytosolic proteins in the absence of adjuvants is able to promote autoimmune injury in the liver [72]. This indicates that, in some ALD patients, the combination of CYP2E1 modification by HERs, impaired CTLA-4 control of T-cell proliferation, and the stimulation of immune system by lipid peroxidation products can lead to the breaking of self-tolerance. Preliminary data shows that high titers of anti-CYP2E1 autoantibodies correlate with the extension of lymphocyte infiltration and the frequency of apoptotic hepatocytes, suggesting that in a subset of ALD autoimmune mechanisms might contribute to tissue injury.

## 4. Possible Role of Adaptive Immunity in the Progression of Alcohol Liver Damage

So far little is known about the mechanisms by which adaptive immunity might contribute to hepatic inflammation in ALD. However, data emerging from experimental models of atherosclerosis indicate that IFN-*γ*, TNF-*α*, and CD40 ligand (CD154) produced by Th-1 CD4^+^ T-cell responsive to antigens in oxidized LDL drive plaque macrophages to produce reactive oxygen species, NO, and proinflammatory cyto/chemokines [73]. Studies in alcohol-fed rodents show that the development of antibodies against lipid-peroxidation-derived epitopes associate with the hepatic expression of proinflammatory cytokines and histological evidence of steatohepatitis [47, 48]. In humans, a prospective survey has evidenced an association between the presence of antibodies toward alcohol-modified hepatocytes and an increased risk of developing alcoholic liver cirrhosis [74]. On the same line, heavy drinkers with lipid-peroxidation-derived antibodies have a 5-fold higher prevalence of elevated plasma TNF-*α* levels than alcohol abusers with these antibodies within the control range [75]. Moreover, in these subjects, the combination of high TNF-*α* and lipid-peroxidation-induced antibodies increases by 11-fold the risk of developing advanced ALD [75]. Interestingly, the combination of steatosis and high titers of antibodies against lipid-peroxidation-derived adducts is an independent predictor of advanced fibrosis/cirrhosis in alcohol-consuming patients with chronic hepatitis C [76]. Further evidence in favor of a possible contribution of oxidative-stress-mediated immunity in sustaining hepatic inflammation comes from studies in nonalcoholic steatohepatitis (NASH). We have observed that antibodies against lipid-peroxidation-derived antigens similar to those detected in ALD, are present in rodent models of NASH [77] as well as in about 40% of adult NAFLD/NASH patients [78] and in 60% of children with NASH [79]. Experiments comparing the development of NASH induced by mice feeding with a methionine/choline deficient (MCD) diet demonstrate that C57BL/6, but not BALB/C, mice have an increased prevalence of liver infiltrating T and B cells and generate antibodies against MDA adducts. Hepatic necroinflammation and the expression of TNF-*α* and IL-12 are significantly higher in C57BL/6 mice than in BALB/C mice. Among MCD-treated mice, a significant positive correlation is also evident between the titers of anti-MDA antibodies and the number of hepatic necroinflammatory foci [80]. In line with these findings, in children with NAFLD, high titers of anti-MDA adduct IgG, but not of other oxidative stress markers, are associated with more severe lobular inflammation and with 13-fold increased risk of overt steatohepatitis [79]. We have observed that in mice with NASH, anti-MDA IgG target specific antigens in hepatic necroinflammatory foci leading to the formation of immunocomplexes. Furthermore, Rensen and coworkers have detected extensive deposition of different complement fractions in liver biopsies form NASH patients that associates with increased hepatocyte apoptosis, granulocyte infiltration and higher liver expression of IL-1*β*, IL-6, and IL-8 mRNAs [81]. This suggests the possibility that complement activation might bridge adaptive and innate immune responses during ALD evolution. Supporting this view, recent observations by Nagy's group demonstrate complement activation within the liver of alcohol-fed mice that leads to increased production of C3a anaphylatoxin [82]. Moreover, mice deficient of C3 and C5 are protected against hepatic injury, while the lack of the complement-regulating protein CD55/DAF exacerbates alcohol hepatotoxicity [82].

## 5. Conclusion and Future Perspectives

In conclusion, data so far obtained indicates that alcohol-induced oxidative modifications of hepatic constituents trigger specific immune responses and, in some conditions, may favor the breaking of the self-tolerance toward liver constituents. The development of adaptive immune responses is likely favored by the capacity of alcohol to stimulate by different ways innate immunity [4–8]. Although still indirect, available evidence also suggests that the development of humoral and cellular immunity may contribute to hepatic inflammation during the evolution of ALD. Nonetheless, during the progression of ALD to cirrhosis, additional mechanisms may be at work. Recent data indicate that the interactions between myofibroblast-like hepatic stellate cells (HSC/MFs), Kupffer cells, and CD4^+^ T-lymphocytes are critical for the regulation of fibrogenic responses [83, 84]. In particular, by expressing adhesion molecules (VCAM-1 and ICAM-1) and chemokines (CCL2, CXCL9/10, CXCL16, and fractalin), HSC/MFs are essential for the liver recruitment of lymphocytes and provide the milieu for their survival and activation [84]. On the other hand, Th-2 cytokines (IL-4 and IL-13) from CD4^+^ T cells directly sustain matrix deposition by HSC/MFs and favor “alternative” M2 activation of macrophages with the production of chemokines (CCL6 and CCL17), IL-10, TGF-*β*, and VEGFs that drive healing responses and angiogenesis [83, 84]. At present, it is not known whether T-cell stimulation by oxidative-stress-derived antigens might promote Th-2 lymphocyte development and M2 macrophage polarization, contributing to fibrogenesis. 

From the clinical point of view, prospective studies are required to dissect out the precise role of immune responses in the progression of human ALD. If supported by further data, the concept that adaptive immunity has a role in alcohol hepatotoxicity might lead to the development of simple immunometric assays to discriminate ALD patients at risk of progressing to hepatitis and/or fibrosis and moreover, the identification of alcohol abusers with a prominent immune or autoimmune component in their hepatic disease that might lead to a targeted use of immune-suppressive therapy in ALD.
